# Metabolic reprogramming and metabolic homeostasis in podocyte injury: novel mechanistic insights and natural product therapies

**DOI:** 10.3389/fcell.2026.1978760

**Published:** 2026-10-09

**Authors:** Zhongjie Qu, Wenqing Ba, Meijing Tang, Wenze Jiang, Mengting Zhu, Keda Lu

**Affiliations:** Department of Nephrology, The Third Affiliated Hospital of Zhejiang Chinese Medical University (Zhongshan Hospital of Zhejiang Province), Hangzhou, Zhejiang, China

**Keywords:** immunometabolism, ion homeostasis, metabolic homeostasis, metabolic reprogramming, natural products, podocyte, traditional Chinese medicine

## Abstract

Podocyte injury and loss are major pathological features of proteinuria and renal dysfunction. Growing evidence has highlighted the contribution of metabolic reprogramming and disrupted metabolic homeostasis to podocyte injury in glomerular disease. Under pathological conditions, alterations in the local microenvironment, including hyperglycemia, lipid overload, mechanical stress, and inflammatory stimuli, can trigger maladaptive metabolic responses in podocytes, characterized by disturbances in glucose, lipid, and amino acid metabolism. These metabolic alterations may be accompanied by disrupted ion homeostasis and crosstalk with innate immune signaling, collectively aggravating the structural and functional damage of podocytes. However, pharmacological options that specifically target metabolic abnormalities in podocytes are limited. Natural products, including traditional Chinese medicine prescriptions, have shown potential for mitigating podocyte injury because of their capacity to act on multiple targets and pathways. A range of natural products may preserve podocyte integrity by correcting mitochondrial dysfunction, reducing oxidative stress, modulating metabolic substrate utilization, restoring ion homeostasis, and attenuating inflammatory responses. This review examines metabolic reprogramming and homeostatic disturbances in podocytes, outlines the mechanisms underlying podocyte metabolic dysfunction, and summarizes how natural products mitigate podocyte injury in glomerular diseases, such as diabetic kidney disease and membranous nephropathy, thereby offering perspectives on metabolic mechanisms and potential therapeutic targets for podocyte-directed interventions.

## 1 Introduction

Podocytes are essential components of the glomerular filtration barrier. With an improved understanding of the biological characteristics and pathological roles of podocytes, podocyte injury and loss are now recognized as major contributors to proteinuria and progressive kidney failure (Meliambro et al., 2024). Previous therapies for podocyte injury have largely relied on renin-angiotensin-aldosterone system blockers and immunosuppressive agents (Miao et al., 2024; Romagnani et al., 2025). Although these treatments can delay disease progression to some extent, precise interventions targeting the intrinsic mechanisms of podocyte injury remain limited. Accumulating evidence indicates that metabolic reprogramming and metabolic homeostasis within podocytes play critical roles in functional impairment and structural injury (Fan et al., 2024; Luo et al., 2024). Metabolic homeostasis refers to a relatively stable state maintained through the dynamic regulation of various biochemical reactions in cells, thereby ensuring an adequate energy supply for cellular processes (Smiles et al., 2024). Cells can dynamically adjust their metabolic and energy-producing pathways to sustain biological functions and adapt to changes in the external environment (Deng et al., 2025; Li J. et al., 2025; Mitchell et al., 2026). This adaptive process is known as metabolic reprogramming, as exemplified by the “Warburg effect” (Deng et al., 2025). Metabolic reprogramming is essential not only for cellular function and survival but also for the development and progression of numerous diseases (Xia et al., 2021; Zhu et al., 2022; Zhang et al., 2025a; Zhang et al., 2025b). At the same time, pathological stimuli can directly affect substrate uptake, synthesis, degradation, or export, thereby leading to metabolic abnormalities. Whether arising passively from pathological stimuli or through active cellular adaptation, metabolic alterations that exceed the regulatory capacity of cells may disrupt metabolic homeostasis. This disruption may manifest as abnormal substrate utilization, impaired energy production, or aberrant metabolite accumulation, ultimately compromising cellular function and survival. In podocytes, metabolic alterations under disease conditions primarily involve glucose, lipid, amino acid, and energy metabolism. Meanwhile, the homeostasis of ions such as iron and calcium is frequently disrupted, further aggravating metabolic disturbances (Yang X. et al., 2024; Zuo F.-w. et al., 2024). In addition, metabolic disturbances can activate cellular inflammatory responses and promote metabolic-immune interactions, thereby contributing to podocyte injury (Shahzad et al., 2022; Hu et al., 2026). Accordingly, targeting metabolic reprogramming and disturbances in podocytes has emerged as a promising therapeutic strategy. Moreover, the renoprotective effects of established therapies, such as SGLT2 inhibitors and GLP-1 receptor agonists, provide indirect clinical support for the therapeutic potential of targeting podocyte metabolic pathways (Bae, 2025; Hu et al., 2026). However, the clinical translation of these strategies faces challenges such as the insufficient protection afforded by single-target interventions and the lack of podocyte-specific delivery systems (Liu C.-P. et al., 2019). Natural products, including traditional Chinese medicine (TCM) prescriptions and naturally derived compounds, are increasingly used in the prevention and treatment of CKD, and are often perceived to have favorable safety profiles (Wang B. et al., 2024; Wang Y. N. et al., 2024; Acquah-Mills et al., 2025; Chen et al., 2025; Liu et al., 2026d). Preclinical studies also suggest that TCM exerts protective effects in various models of podocyte injury (Miao et al., 2022; Chen et al., 2024b; Liu Y. et al., 2024; Cai et al., 2025; Gao et al., 2025). However, studies investigating the podocyte-protective effects of TCM through the regulation of metabolism-related targets have not yet been systematically synthesized. Therefore, this review focuses on podocyte metabolism, summarizes the mechanisms through which metabolic reprogramming and metabolic disturbances contribute to podocyte injury, including alterations in glucose, lipid, and amino acid metabolism, ion homeostasis, and immunometabolism, and discusses recent advances and future prospects of TCM-based strategies targeting podocyte metabolism. While a recent review comprehensively examined the mechanisms of metabolic reprogramming in podocytes and related modern pharmacological interventions (Hu et al., 2026), our focus here is to provide a systematic and critical evaluation of natural products as a therapeutic strategy for modulating these pathways and to consider their translational potential.

Literature search and selection criteria: This narrative review was based on literature searches conducted in PubMed and Web of Science, covering the period from the inception of each database to March 2026. The keywords included “podocyte,” “traditional Chinese medicine,” “Chinese herbal drugs,” “herbal medicine,” “plant extracts,” and “medicinal plants.” To support the mechanistic sections, we also searched and evaluated literature across the principal mechanistic domains covered in this review, including glucose, lipid, and amino acid metabolism, iron homeostasis and ferroptosis, calcium homeostasis, and immunometabolism. Additional studies were identified by manually screening the reference lists of relevant articles. We included original studies, including animal studies, cell-based studies, and clinical studies, as well as reviews published in peer-reviewed journals. For the section on natural products, studies were included if they were relevant to any of these domains in podocytes. Conference abstracts and theses were excluded.

## 2 Podocytes sense and respond to changes in the surrounding microenvironment

Podocytes, the glomerular basement membrane (GBM), and glomerular endothelial cells constitute the glomerular filtration barrier. Through their highly specialized cellular architecture, podocytes maintain selective filtration of macromolecules within the glomerulus. They are firmly anchored to GBM through large primary processes and interdigitating secondary foot processes (Garg, 2018). Filtration slits are formed between the secondary foot processes of adjacent podocytes and are bridged by the slit diaphragm, which contains transmembrane proteins such as nephrin and podocin (Lu et al., 2017). Additionally, podocytes preserve the functional integrity of neighboring endothelial cells by secreting vascular endothelial growth factor and other factors, thereby providing essential support for the trilaminar glomerular filtration structure (Sison et al., 2010). Podocytes are continuously exposed to shear stress generated by glomerular capillary blood flow, mechanical strain arising from filtration pressure, and glucose, lipids, amino acids, and various metabolites in the filtrate. They express diverse mechanosensors, nutrient-sensing receptors, and innate immune receptors that enable them to detect changes in their dynamic microenvironment (Haydak and Azeloglu, 2024). In response to these complex microenvironmental stimuli, podocytes undergo a series of metabolic adaptations that regulate nutrient uptake and ion balance to preserve functional homeostasis, involving glucose, lipid, and amino acid metabolism, ion homeostasis, and immunometabolism. When these sensing or adaptive mechanisms become dysregulated, microenvironmental signals may promote actin filament depolymerization and cytoskeletal remodeling, resulting in foot process effacement, podocyte detachment, epithelial–mesenchymal transition, and various forms of programmed cell death, including apoptosis, pyroptosis, and ferroptosis (Yang H. et al., 2024) (Figure 1). Moreover, because the structural organization of the podocyte cytoskeleton is incompatible with mitotic spindle formation, forced cell cycle entry frequently results in podocyte loss and death through mitotic catastrophe (Liapis et al., 2013; Zhang and Guo, 2025). This restricted regenerative capacity means that once podocyte loss exceeds a critical threshold, irreversible glomerular injury may develop, followed by persistent proteinuria and a progressive decline in the glomerular filtration rate (Wharram et al., 2005).

**FIGURE 1:**
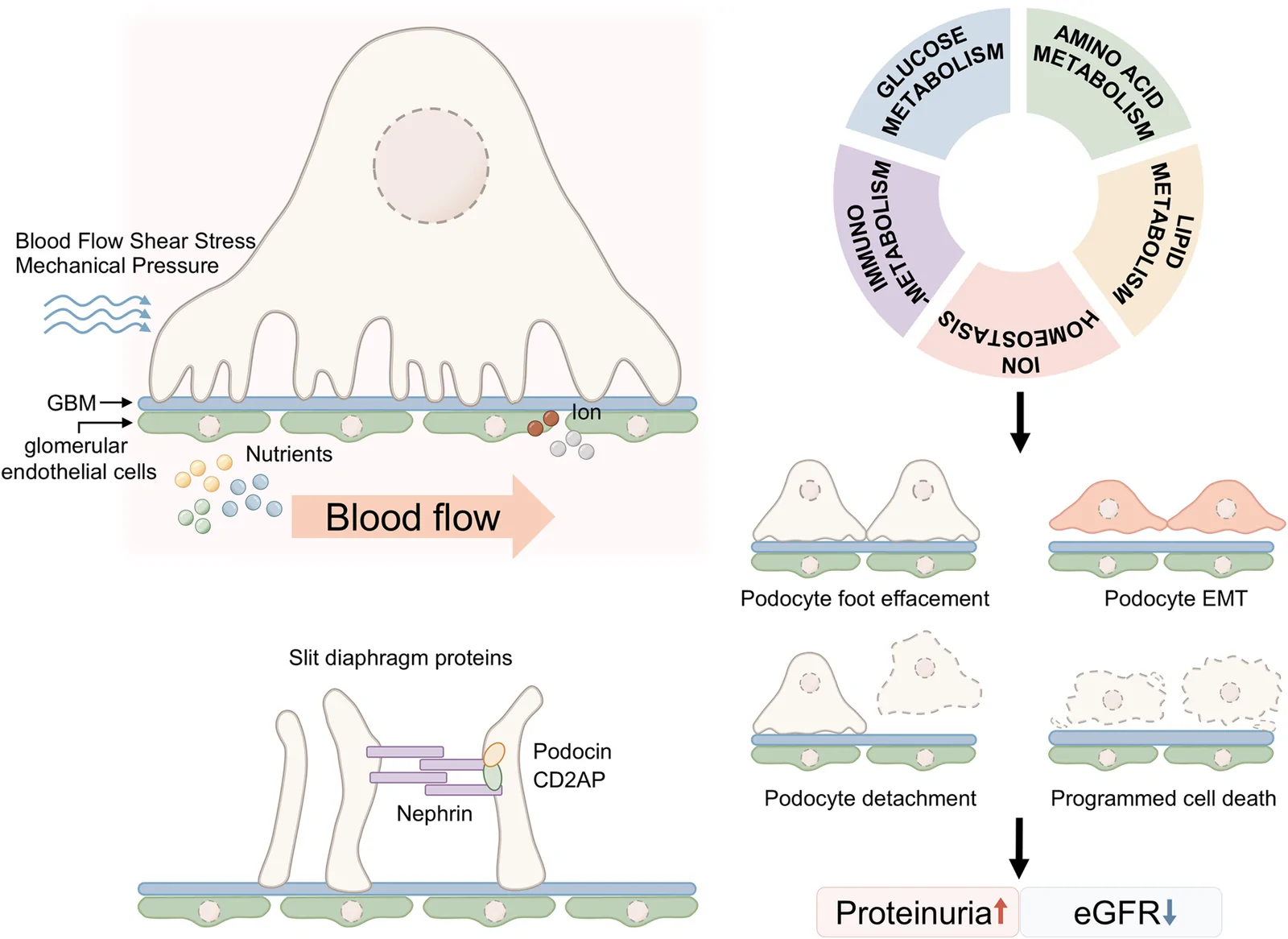
Podocyte structure and pathological processes underlying podocyte injury. The glomerular filtration barrier consists of glomerular endothelial cells, GBM, and podocytes. Interdigitating podocyte foot processes anchor the GBM and form filtration slits. Slit diaphragm proteins, including nephrin, podocin, and CD2AP, contribute to maintaining the filtration barrier’s selective permeability. Podocytes sense signals from the surrounding microenvironment, including blood flow-induced shear stress, mechanical pressure, nutrient levels, and ion levels. Under pathological conditions, disturbances in glucose, amino acid, and lipid metabolism, ion homeostasis, and immunometabolism contribute to podocyte injury, leading to foot process effacement, podocyte detachment, EMT, and programmed cell death, ultimately resulting in proteinuria and a decline in eGFR. CD2AP, CD2‐associated protein; eGFR, estimated glomerular filtration rate; EMT, epithelial–mesenchymal transition; GBM, glomerular basement membrane.

## 3 Metabolic reprogramming and metabolic homeostasis in podocytes

### 3.1 Glucose metabolism

There is no clear consensus on the predominant energy source for podocytes under physiological conditions. An early study suggested that energy production in cultured mouse podocytes primarily depends on mitochondrial function (Abe et al., 2010). Another study suggested that both glycolysis and mitochondrial oxidative phosphorylation contribute to energy metabolism in podocytes, whereas the ATP supply in foot processes may depend predominantly on glycolysis (Ozawa et al., 2015). Brinkkoetter et al. proposed that podocytes primarily rely on anaerobic glycolysis for energy production and found that podocyte-specific knockout of key genes involved in mitochondrial fission, biogenesis, and transcription did not cause obvious phenotypic abnormalities in mouse podocytes under physiological conditions (Brinkkoetter et al., 2019). However, another study found that both glycolysis and mitochondrial oxidative phosphorylation are enhanced during podocyte differentiation to accommodate increased energy demands, and that energy production in mature podocytes may shift from anaerobic glycolysis to mitochondrial oxidative phosphorylation (Yuan et al., 2020).

Similarly, changes in podocyte energy metabolism under pathological conditions are controversial. Some studies have reported that both glycolysis and mitochondrial oxidative phosphorylation are reduced in podocytes under high glucose conditions (Qi et al., 2017). Excess glucose entering the mitochondria increases the burden on the tricarboxylic acid cycle and electron transport chain, causing excessive electron leakage and substantial production of reactive oxygen species (ROS), while impairing the activity of mitochondrial respiratory chain complexes and consequently reducing respiratory efficiency and ATP production (Jha et al., 2016). Excessive ROS also induces oxidative stress in podocytes, causing pyruvate kinase M2 (PKM2) to dissociate from its highly active tetrameric form into a less active dimer, thereby reducing glycolytic flux and aggravating mitochondrial dysfunction. The PKM2 activator TEPP-46 can reverse these alterations and improve the renal pathology in diabetic mice (Qi et al., 2017). Angiotensin II may suppress glycolysis and ATP production by reducing PKM2 expression and activity (Chen Z. et al., 2023), whereas Smad4 may reduce the active tetrameric form of PKM2 and impair glycolysis and OXPHOS through interaction with PKM2 and ATPIF1 (Li et al., 2020c). However, another study reported impaired oxidative phosphorylation accompanied by a metabolic shift toward glycolysis during the *in vitro* differentiation of human podocytes exposed to 20 mM glucose (Imasawa et al., 2017). Similarly, in a study of podocyte injury in idiopathic membranous nephropathy, sPLA2-IB stimulated PLA2R on podocytes and activated the mTOR/HIF-1α signaling axis, thereby increasing PKM2 and lactate dehydrogenase A (LDHA) expression and enhancing glycolysis while suppressing the TCA cycle, ultimately resulting in an abnormal podocyte energy state (Bao et al., 2025). Overall, the mode of energy production in podocytes may vary depending on disease type, cell source, differentiation status, and culture conditions, and the available evidence remains insufficient to explain all physiological and pathological contexts in terms of a single metabolic pathway.

Lactate is an important product of glycolysis, and its metabolism is frequently disrupted in podocytes under hyperglycemic conditions. Elevated intracellular lactate levels in podocytes can promote histone and non-histone lactylation, inducing epithelial–mesenchymal transition and suppressing autophagic flux, ultimately leading to podocyte apoptosis (Fan et al., 2025; Zheng et al., 2025). However, studies examining the upstream mechanisms responsible for elevated lactate levels have reported inconsistent findings. Audzeyenka et al. found that high glucose reduced lactate dehydrogenase activity and LDHA expression in podocytes, accompanied by impaired glycolytic flux and abnormal intracellular accumulation of lactate and glycogen, which subsequently induced insulin-dependent impairment of glucose uptake and disruption of foot process architecture. High glucose also decreased monocarboxylate transporter 1 expression and increased monocarboxylate transporter 4 expression, while increasing the levels of both transporters in the cell membrane (Audzeyenka et al., 2025). In contrast, Zheng et al. observed that chronic high-glucose stimulation did not significantly alter the expression of LDHA or monocarboxylate transporter 4 in the podocytes. They proposed that abnormal lactate accumulation was more likely attributable to the upregulation of monocarboxylate transporter 2 and reduced pyruvate dehydrogenase activity, with the discrepancy potentially reflecting differences in cell models, duration of high-glucose exposure, and other experimental conditions (Zheng et al., 2025). Therefore, the dynamic alterations in podocyte glycolytic activity and the mechanisms underlying lactate accumulation under hyperglycemic conditions require further clarification.

Glucose metabolism is closely linked to mitochondrial homeostasis, and mitochondrial integrity is essential for maintaining podocyte structure and function. Under physiological conditions, the mitochondria maintain a dynamic equilibrium through continuous fusion and fission. This process is regulated by dynamin-related protein 1 (Drp1), mitofusin 1 (Mfn1), mitofusin 2 (Mfn2), optic atrophy 1 (Opa1), and other related proteins (Gujarati et al., 2020). In parallel, peroxisome proliferator-activated receptor-γ coactivator-1α (PGC-1α), a key regulator of mitochondrial biogenesis, contributes to mitochondrial homeostasis through interactions with several transcription factors. PGC-1α activity can be enhanced through AMP-activated protein kinase (AMPK)-mediated phosphorylation and silent information regulator sirtuin 1 (SIRT1)-mediated deacetylation (Fontecha-Barriuso et al., 2020). High glucose levels can activate ROCK1 and promote Drp1 phosphorylation at Ser600, resulting in increased mitochondrial fission (Wang et al., 2012; Galvan et al., 2019). Meanwhile, high glucose downregulates mitochondrial fusion-related proteins such as Mfn1, Mfn2, and Opa1 (Audzeyenka et al., 2021), thereby disturbing the balance between mitochondrial fission and fusion. Simultaneously, PGC-1α expression is reduced in podocytes under high-glucose conditions, resulting in the downregulation of nuclear respiratory factor 1 (Nrf1) and transcription factor A, mitochondrial (TFAM), which subsequently suppresses mitochondrial DNA (mtDNA) replication and mitochondrial biogenesis (Imasawa et al., 2017; Audzeyenka et al., 2021). Beyond high glucose conditions, increased phosphorylation of Drp1 at the Ser616 site was observed in podocytes from patients with lupus nephritis (LN) (Ye et al., 2024). Collectively, these alterations change mitochondrial morphology and reduce mitochondrial abundance, further promoting excessive ROS generation and impairing mitochondrial oxidative phosphorylation.

Disruption of glucose metabolic homeostasis is also reflected by the aberrant activation of alternative glucose-processing pathways, such as the polyol pathway. Under physiological conditions, only a small proportion of glucose enters the polyol pathway, in which glucose is reduced to sorbitol and subsequently oxidized to fructose through reactions involving aldose reductase and nicotinamide adenine dinucleotide phosphate (NADPH) (Tang et al., 2012). Under high-glucose conditions, the intracellular glucose load in podocytes increases. Excess glucose is diverted into the polyol pathway, resulting in enhanced sorbitol production and abnormal intracellular accumulation, ultimately promoting oxidative stress and disturbing glucose metabolic homeostasis (Lagies et al., 2019).

Ultimately, despite abundant glucose availability, severe ATP depletion compromises the ability of podocytes to sustain dynamic cytoskeletal rearrangements, leading to foot-process effacement, hypertrophy, and podocyte detachment. In addition, excessive accumulation of sorbitol and lactate further promotes podocyte apoptosis, contributing to proteinuria and the progressive loss of glomerular filtration function. In summary, disturbances in glucose metabolic homeostasis in podocytes involve alterations in glycolysis and mitochondrial oxidative phosphorylation, regardless of whether these alterations constitute actively regulated metabolic reprogramming. Although the direction of these alterations varies across experimental conditions, coordination between these two energy-producing pathways contributes to maintaining podocyte energy supply and physiological function. Therefore, specifically targeting metabolic regulators to maintain appropriate glycolytic and mitochondrial oxidative phosphorylation levels in podocytes may be crucial for preventing podocyte injury.

### 3.2 Amino acid metabolism

Amino acid metabolism plays an important role in renal disease (He et al., 2025; Zhang et al., 2026). As a fundamental component of cellular metabolism, amino acid metabolism participates in energy-generating processes, such as glycolysis and the tricarboxylic acid cycle, and in the synthesis of proteins, nucleic acids, and lipids. Amino acid metabolism is an emerging area of research, with recent studies suggesting that dysregulated amino acid metabolism is associated with podocyte injury. When glycolysis is impaired, podocytes compensate for the resulting cellular energy deficit by enhancing ornithine catabolism. Ornithine decarboxylase 1 and arginase 2 are upregulated in podocytes, resulting in excessive putrescine production and overactivation of the Rheb-mTORC1 signaling axis, which subsequently promotes cytoskeletal remodeling and cell migration (Luo et al., 2022). In addition, defective branched-chain amino acid (BCAA) catabolism may be a key metabolic event contributing to podocyte injury. A recent study identified BCAA catabolic defects in podocytes from patients with diabetic kidney disease (DKD) and db/db mice. Abnormal accumulation of BCAAs and their intermediate metabolites, branched-chain α-keto acids (BCKAs), not only impairs glucose oxidative phosphorylation but also redirects glucose metabolism toward serine biosynthesis and folate metabolism, potentially resulting in energy deficiency and cellular dysfunction. This metabolic disturbance might be associated with BCAA-induced depolymerization and inactivation of PKM2 (Zhao et al., 2025). In addition to BCAAs, dysregulated glutamine metabolism may contribute to energy deficiency in podocytes. Under high-glucose exposure, reduced Liver Receptor Homologue-1 activity leads to the downregulation of glutaminase 2 expression and impaired glutamine catabolism, ultimately decreasing mitochondrial ATP production and promoting podocyte apoptosis (Hu et al., 2023). These metabolic abnormalities involve not only the shifts toward amino acid-related metabolic pathways, but also the disruption of amino acid pools and the accumulation of metabolic intermediates that may drive the transition of podocytes from metabolic compensation to structural disruption and cell death. Therefore, regulating amino acid metabolism and restoring metabolic homeostasis in podocytes may provide a novel strategy for preserving the glomerular filtration barrier.

### 3.3 Lipid metabolism

Aberrant lipid metabolism plays a critical role in the development and progression of various renal diseases (Liu H. J. et al., 2023; Wang et al., 2023b; Li et al., 2024c; Wang Y. et al., 2024). Under physiological conditions, podocytes maintain lipid metabolic homeostasis by balancing lipid uptake, efflux, synthesis, and oxidation (Li et al., 2024b). Fatty acids enter podocytes through transporters such as CD36 and fatty acid transport protein 4 (FATP4) (Mitrofanova et al., 2023) and serve as important substrates for mitochondrial energy metabolism, contributing to ATP production via fatty acid oxidation (FAO) (Brinkkoetter et al., 2019). Acetyl-CoA can be converted into fatty acids through the catalytic activities of acetyl-CoA carboxylase 1 (ACC1) and fatty acid synthase (Kayampilly et al., 2022). Cholesterol is primarily taken up through the low-density lipoprotein receptor (LDLR) and exported through the ATP-binding cassette transporter A1 (ABCA1) (Mitrofanova et al., 2023). Cholesterol contributes to the maintenance of cell membrane architecture, lipid raft organization, and slit diaphragm function (Fornoni et al., 2014). When intracellular fatty acid or cholesterol levels remain elevated, podocytes can esterify these lipids and sequester them in lipid droplets, thereby limiting the burden of free lipids (Schelling, 2022). Members of the peroxisome proliferator-activated receptor (PPAR) family, including PPARα, PPARδ, and PPARγ, play essential roles in lipid metabolic homeostasis by promoting oxidative lipid catabolism and regulating lipid storage within lipid droplets (Montaigne et al., 2021; Carrasco et al., 2023).

However, in diseases such as diabetes and obesity, this balance may be disrupted by stimuli, including hyperglycemia and free fatty acids (Luo et al., 2024). Studies have demonstrated that hyperglycemia alters the expression of several proteins involved in lipid transport. Downregulation of acyl-protein thioesterase 1 promotes the abnormal accumulation of palmitoylated CD36 on the plasma membrane (Wang J. et al., 2025), whereas a deficiency of dedicator of cytokinesis 5 enhances CD36 activity by upregulating liver X receptor α (LXRα) expression (Qu et al., 2024). In addition, free palmitate can increase CD36 expression in podocytes (Hua et al., 2015), whereas the paracrine factor VEGF-B upregulates FATP4 expression under hyperglycemic conditions (Falkevall et al., 2017), collectively enhancing fatty acid uptake. Activation of G-protein-coupled receptor 43 may increase LDLR-mediated cholesterol uptake through upregulation of extracellular signal-regulated kinase 1/2 (ERK1/2) (Lu et al., 2022). Together, these alterations increase lipid influx and predispose the podocytes to lipid overload. Meanwhile, the upregulation of coiled-coil domain-containing 92 promotes ABCA1 degradation (Zuo F.-w. et al., 2024; Zuo F. et al., 2024), impairing cholesterol efflux and further increasing cholesterol accumulation in podocytes. In addition to altered lipid transport, impaired FAO is another major mechanism underlying podocyte lipid accumulation. Under hyperglycemic conditions, the activation of Rho-associated coiled-coil containing protein kinase 2 suppresses the PPARα signaling pathway (Matoba et al., 2022), while the upregulation of the ubiquitin ligase TRIM63 promotes PPARα ubiquitination and degradation, resulting in the downregulation of FAO-related genes and impaired mitochondrial fatty acid oxidation (Chen Q. et al., 2023). Concurrent upregulation of junctional adhesion molecule-like protein (JAML) suppresses SIRT1 expression and subsequently increases the expression of lipogenic enzymes, such as ACC1, through sterol regulatory element-binding protein 1 (SREBP1) (Fu et al., 2020). In addition, fat mass- and obesity-associated protein can reduce m6A modification of ACC1 mRNA, thereby increasing its stability and promoting fatty acid synthesis (Chang et al., 2025), which further aggravates lipid overload in podocytes. Once the lipid droplet storage capacity is exceeded, excess free lipids exert lipotoxic effects and contribute to podocyte injury. Furthermore, hyperglycemia disrupts cardiolipin metabolism and promotes the abnormal accumulation of lipotoxic species, such as ceramides. Cardiolipin accumulation and abnormal remodeling can impair mitochondrial function (Ducasa et al., 2019; Hao et al., 2024), whereas CerS6-derived ceramides promote mtDNA release, triggering immune and inflammatory responses (Zhu et al., 2025). Ultimately, excessive lipid accumulation in podocytes contributes to membrane disruption, increased ROS production, endoplasmic reticulum stress, impaired autophagy, and enhanced apoptosis (Schelling, 2022). Whether altered lipid metabolism and lipid accumulation are passive consequences of metabolic dysfunction or adaptive responses to increased energy demand remains unclear. Nevertheless, selectively targeting key regulators of lipid uptake, efflux, synthesis, and oxidation in podocytes to restore lipid metabolic homeostasis and ameliorate proteinuria is a potential therapeutic strategy.

### 3.4 Ion homeostasis

Iron and calcium are essential intracellular inorganic ions whose homeostasis influences podocyte structure and function by regulating metabolic enzyme activity, mediating oxidative stress, and modulating intracellular signaling, thereby serving as key regulatory nodes in the metabolic homeostasis of podocytes.

#### 3.4.1 Iron and ferroptosis

Under physiological conditions, circulating Fe^3+^ binds to transferrin to form a transferrin–Fe^3+^ complex, which subsequently interacts with transferrin receptors on the cell membrane and is internalized through endocytosis. Within the cell, Fe^3+^ is reduced to Fe^2+^, which either remains in the cytosol as part of the labile iron pool or binds to ferritin for stable storage (Li et al., 2020b). Meanwhile, intracellular iron levels are regulated by the iron regulatory protein-iron responsive element system. The iron stored in ferritin can also be released through NCOA4-mediated ferritinophagy (Bayır et al., 2023). However, under pathological conditions, the specific roles of these iron-handling mechanisms in podocytes warrant further investigation.

When intracellular Fe^2+^ exceeds the storage capacity of ferritin, excess free iron generates large amounts of ROS through the Fenton reaction, resulting in lipid peroxidation and ultimately triggering ferroptosis (Stockwell, 2022). The hallmarks of ferroptosis, including the accumulation of ROS and lipid peroxides, have been observed in pathological conditions such as DKD and LN. The System Xc^−^/GSH/GPX4 axis is the best-characterized regulatory pathway of ferroptosis. The cystine/glutamate antiporter system (System Xc^−^) imports extracellular cystine while exporting intracellular glutamate, thereby maintaining glutamate and cysteine homeostasis and supporting glutathione (GSH) synthesis. Glutathione peroxidase 4 (GPX4) utilizes GSH to limit lipid peroxidation damage in podocytes (Liu Y. et al., 2023). In addition, several GPX4-independent pathways, including the FSP1/CoQ10 and GCH1/BH4 axes, act in concert to suppress ferroptosis and preserve metabolic homeostasis (Doll et al., 2019; Kraft et al., 2020). Nuclear factor erythroid 2-related factor 2 (Nrf2) is a key regulator of ferroptosis because it controls multiple processes involving iron metabolism, GSH synthesis, and NADPH regeneration (Dodson et al., 2019). In DKD, multiple signaling pathways disrupt the System Xc^−^/GSH/GPX4 axis and disturb cellular homeostasis. For example, high fructose upregulates mitochondrial single-stranded DNA-binding protein 1 in podocytes, which activates DNA-dependent protein kinase and promotes the nuclear translocation of phosphorylated p53, thereby suppressing the transcription of solute carrier family 7 member 11 (SLC7A11), the functional subunit of System Xc^−^ (Wu et al., 2022). Recent studies have shown that phosphoglycerate dehydrogenase deficiency under hyperglycemic conditions promotes ubiquitin-mediated degradation of Y-box binding protein 1, thereby reducing SLC7A11 mRNA stability and expression (Wang Y. et al., 2025). Upregulation of MAPK14 suppresses the expression of both SLC7A11 and GPX4 (Qiu et al., 2026). In addition, hyperglycemia downregulates specificity protein 1 (SP1), resulting in reduced peroxiredoxin 6 transcription and consequently aggravating oxidative stress and ferroptosis in podocytes (Zhang et al., 2021). In LN, the downregulation of OTUB1, a deubiquitinating enzyme, reduces SLC7A11 expression, thereby contributing to podocyte injury (Liu C. et al., 2024). Upregulation of CYBB, which encodes NOX2, enhances ROS generation and promotes lipid peroxidation by reducing GSH, GPX4, and SLC7A11 levels, thereby inducing ferroptosis in podocytes (Wang et al., 2026). Furthermore, Kang et al. found that the fatty acid elongase Elovl7 is upregulated in injured podocytes, promoting the accumulation of long-chain polyunsaturated fatty acids and increasing podocyte susceptibility to ferroptosis, thereby aggravating ferroptotic injury and podocyte loss (Kang et al., 2025).

#### 3.4.2 Calcium (Ca^2+^)

Ca^2+^ is a critical intracellular messenger that plays an essential role in regulating podocyte growth and development, autophagy, gene transcription, and cytoskeletal dynamics (Carney, 2021; Sukumaran et al., 2021). Transient receptor potential channel C6 (TRPC6) is a key channel located in the slit diaphragm that participates in the regulation of Ca^2+^ signaling (Hall et al., 2020). In addition, the endoplasmic reticulum and mitochondria coordinate Ca^2+^ homeostasis through mitochondria-associated endoplasmic reticulum membranes (MAMs) (Barazzuol et al., 2021).

Under pathological conditions such as hyperglycemia, TRPC6 can be aberrantly activated by mechanical stimulation, angiotensin II, purinergic signaling, and increased local ROS production, resulting in excessive Ca^2+^ influx (Staruschenko et al., 2023). Elevated intracellular Ca^2+^ levels activate calcineurin and Ca^2+^/calmodulin-dependent protein kinase 4 (CaMK4). Calcineurin dephosphorylates synaptopodin, whereas CaMK4 phosphorylates the synaptopodin-associated scaffolding protein 14-3-3, disrupting the interaction between synaptopodin and 14-3-3 and promoting synaptopodin release and degradation. In parallel, CaMK4 modulates the activity of Rho-family small GTPases, including Rac1 and RhoA, leading to cytoskeletal remodeling, apoptosis, and proteinuria (Faul et al., 2008; Maeda et al., 2018; Asano-Matsuda et al., 2021). In addition, calcineurin can further increase TRPC6 expression through nuclear factor of activated T cells (NFAT) signaling (Nijenhuis et al., 2011), while the downregulation of synaptopodin can increase TRPC6 localization at the cell membrane, thereby aggravating foot process effacement and podocyte injury (Yu et al., 2016). TRPC6 activation also promotes Calpain-1 activity, resulting in the degradation of cytoskeleton-associated anchoring proteins such as Talin-1 and consequent disruption of the podocyte cytoskeleton (Verheijden et al., 2018). It also induces Drp1 phosphorylation via the Calpain-1/CDK5 axis, causing excessive mitochondrial fragmentation and increasing ROS production (Yu et al., 2022), ultimately leading to podocyte injury and apoptosis. Moreover, dysregulated endoplasmic reticulum–mitochondrial Ca^2+^ transfer represents another important component of disturbed Ca^2+^ homeostasis. High glucose levels downregulate transient receptor potential cation channel subfamily V member 1 (TRPV1) expression in podocytes and enhance endoplasmic reticulum–mitochondrial Ca^2+^ transfer, resulting in mitochondrial Ca^2+^ overload, mitochondrial dysfunction, and podocyte injury (Wei et al., 2020).

In summary, restoring ion homeostasis in podocytes is an important strategy for preserving podocyte function and ameliorating proteinuria and renal dysfunction. The restoration of iron homeostasis, reinforcement of ferroptosis defense pathways, and re-establishment of intracellular Ca^2+^ homeostasis in podocytes without disrupting physiological iron metabolism and Ca^2+^ signaling requires further investigation.

### 3.5 Immunometabolism

Immunometabolism in this review refers to the dynamic interplay between podocyte metabolic stress and immune activation. This usage is distinct from the classical concept of immunometabolism, which mainly describes changes in metabolic pathways within immune cells that regulate their function (O'Neill et al., 2016). Metabolic stress activates innate immune receptors in podocytes, whereas a persistent inflammatory milieu further perturbs metabolic homeostasis, collectively promoting structural damage and podocyte death. Similar to immune cells, podocytes express a range of innate immune receptors, including Toll-like receptors (TLRs), NOD-like receptors (NLRs), and RIG-I-like receptors (RLRs), which recognize pathogen-associated molecular patterns (PAMPs) and damage-associated molecular patterns (DAMPs) and initiate appropriate immune responses to maintain physiological function (Banas et al., 2008; Leemans et al., 2014). Dysregulated TLR signaling and NLRP3 inflammasome activation in podocytes have been implicated in DKD, LN, and other glomerular diseases. For instance, high glucose levels and extracellular free fatty acids, such as palmitate, can activate TLR4 and the NLRP3 inflammasome, thereby inducing inflammatory responses in podocytes (Xu et al., 2021; Li Z. et al., 2026). Additionally, accumulated metabolic intermediates, mtDNA, and excessive ROS within podocytes can activate transcription factors, such as NF-κB, and stimulate the NLRP3 inflammasome, increasing the release of inflammatory mediators and disrupting the podocyte cytoskeleton (Ma et al., 2014; Luo et al., 2024). Furthermore, CD36 promotes podocyte injury in LN through activation of the NLRP3 inflammasome (Lv et al., 2022), suggesting that CD36 may represent a molecular link between metabolic dysregulation and innate inflammatory responses. The cyclic GMP-AMP synthase–stimulator of IFN genes (cGAS–STING) pathway also plays a crucial role in linking metabolic disturbances to immune activation. Metabolic disturbances can induce mitochondrial injury, and mtDNA released into the cytoplasm can activate the cGAS–STING pathway, promoting the release of cytokines such as IL-6 and TNF-α through NF-κB and TBK1 signaling, and inducing podocyte injury (Mitrofanova et al., 2022; Zang et al., 2022). Moreover, Yang et al. found that activated STING can upregulate the NLRP3 inflammasome through the TBK1/NF-κB signaling pathway, which subsequently activates Caspase-1, mediates the release of cytokines such as IL-1β and IL-18, and promotes podocyte pyroptosis (Yang et al., 2023). Evidence from genetic knockout models indicates that NLRP3 deletion confers protection against DKD, whereas Caspase-1 deletion provides only partial protection, suggesting that NLRP3 may also contribute to podocyte injury through Caspase-1-independent mechanisms (Shahzad et al., 2022). Immune activation can reshape metabolic processes. For example, the NLRP3 inflammasome can exacerbate mitochondrial injury and thereby aggravate energy metabolic dysfunction, whereas inflammatory mediators can increase the expression of LDLR, SREBP2, and CD36, enhancing lipid uptake and synthesis in podocytes and promoting lipid accumulation (Zhang et al., 2015; Yang et al., 2017). Inhibition of NLRP3 can reduce lipid accumulation in podocytes by downregulating SREBP1/SREBP2 and upregulating ABCA1 (Wu et al., 2021). Therefore, targeting innate immune receptors, such as TLRs and the NLRP3 inflammasome, and transcription factors, such as NF-κB, to suppress chronic inflammation and restore metabolic homeostasis, represents another potential strategy for preserving podocyte viability.

As described above, the regulatory mechanisms underlying metabolic reprogramming and metabolic homeostasis in podocytes are highly complex, with multiple processes occurring simultaneously and extensive crosstalk among different metabolic pathways. Although the interactions and feedback mechanisms among these pathways have not been fully elucidated, existing evidence suggests that mitochondrial dysfunction and ROS may serve as important convergent nodes. Under pathological conditions, mitochondrial function can be impaired by metabolic disturbances. Lipid overload can increase ROS production and impair mitochondrial function, while cardiolipin accumulation and aberrant remodeling can compromise mitochondrial structure and function. Disrupted Ca^2+^ homeostasis can also promote excessive mitochondrial fission. Conversely, mitochondrial dysfunction can alter the utilization of metabolic substrates such as glucose, lipids, and amino acids, leading to insufficient energy supply and potentially promoting the accumulation of lipids or other metabolic intermediates. Oxidative stress is also closely associated with podocyte metabolism. ROS production can be influenced by pathological stimuli, while mitochondrial dysfunction can also result in excessive ROS generation. When ROS levels exceed the antioxidant capacity of podocytes, excessive ROS can further exacerbate mitochondrial dysfunction and lipid metabolic dysregulation. In addition, ROS can activate NF-κB signaling and the NLRP3 inflammasome, whereas CerS6-derived ceramides can promote mtDNA release. The released mtDNA subsequently activates the cGAS–STING signaling pathway, thereby linking metabolic stress to innate immune activation (Figure 2).

**FIGURE 2:**
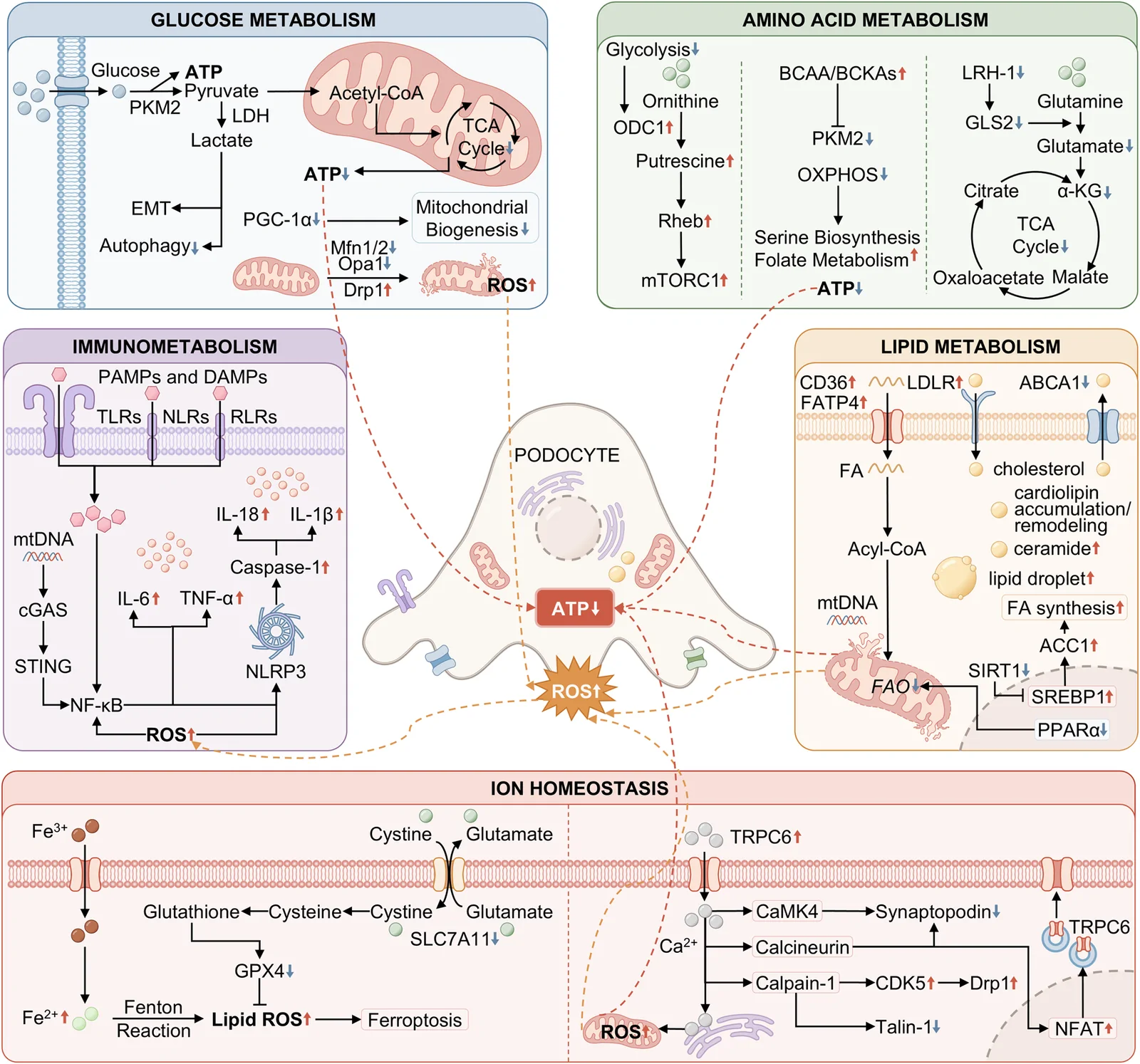
Metabolic reprogramming and metabolic disturbances in podocytes. Schematic representation of metabolic, ionic, and innate immune alterations in injured podocytes. Alterations in PKM2 activity and glycolysis are context dependent. Therefore, no fixed direction is assigned to these changes in the figure. Mitochondrial oxidative phosphorylation is generally impaired under the pathological conditions. Downregulation of PGC-1α suppresses mitochondrial biogenesis, and increased Drp1 together with decreased Mfn1, Mfn2, and Opa1 disrupts mitochondrial dynamics, resulting in excessive ROS production and reduced ATP generation. Lactate accumulation induces EMT while suppressing autophagic flux. Enhanced ornithine catabolism, accompanied by ODC1 upregulation, promotes putrescine production and activates Rheb-mTORC1 signaling. The accumulation of BCAAs and BCKAs promotes PKM2 depolymerization and inactivation and redirects glucose metabolism toward serine biosynthesis and folate metabolism. Glutamine catabolism is suppressed through downregulation of the LRH-1/GLS2 pathway. Lipid uptake mediated by CD36, FATP4, and LDLR is increased, whereas ABCA1-mediated cholesterol efflux is reduced. Downregulation of PPARα impairs FAO, whereas reduced SIRT1 promotes fatty acid synthesis through SREBP1/ACC1. The System Xc^−^/GSH/GPX4 antioxidant system is impaired, and reduced GPX4 and SLC7A11 expression promotes lipid peroxidation and ferroptosis. TRPC6-mediated Ca^2+^ influx is increased and is associated with synaptopodin and Talin-1 downregulation, CDK5/Drp1-mediated mitochondrial fission, ROS accumulation, and apoptosis. NFAT upregulation further increases TRPC6 expression. Activation of the cGAS-STING pathway, NF-κB, and NLRP3 inflammasome increases IL-6 and TNF-α production and promotes caspase-1-dependent release of IL-1β and IL-18. ABCA1, ATP-binding cassette transporter A1; ACC1, acetyl-CoA carboxylase 1; BCAA, branched-chain amino acid; BCKAs, branched-chain α-keto acids; CaMK4, Ca^2^^+^/calmodulin-dependent protein kinase 4; CDK5, cyclin-dependent kinase 5; cGAS, cyclic GMP-AMP synthase; DAMPs, damage-associated molecular patterns; Drp1, dynamin-related protein 1; EMT, epithelial–mesenchymal transition; FA, fatty acid; FAO, fatty acid oxidation; FATP4, fatty acid transport protein 4; GLS2, glutaminase 2; GPX4, glutathione peroxidase 4; IL, interleukin; LDH, lactate dehydrogenase; LDLR, low-density lipoprotein receptor; LRH-1, liver receptor homologue-1; Mfn1, mitofusin 1; Mfn2, mitofusin 2; mtDNA, mitochondrial DNA; mTORC1, mechanistic target of rapamycin complex 1; NF-κB, nuclear factor-κB; NFAT, nuclear factor of activated T cells; NLRs, NOD-like receptors; NLRP3, NOD-like receptor family pyrin domain-containing protein 3; ODC1, ornithine decarboxylase 1; Opa1, optic atrophy 1; OXPHOS, oxidative phosphorylation; PAMPs, pathogen-associated molecular patterns; PGC-1α, peroxisome proliferator-activated receptor-γ coactivator-1α; PKM2, pyruvate kinase M2; PPAR, peroxisome proliferator-activated receptor; Rheb, Ras homolog enriched in brain; RLRs, RIG-I-like receptors; ROS, reactive oxygen species; SIRT1, sirtuin 1; SLC7A11, solute carrier family 7 member 11; SREBP1, sterol regulatory element-binding protein 1; STING, stimulator of IFN genes; TCA cycle, tricarboxylic acid cycle; TLRs, Toll-like receptors; TNF-α, tumor necrosis factor-α; TRPC6, transient receptor potential channel C6; α-KG, α-ketoglutarate.

## 4 Natural products improve podocyte injury by regulating metabolic reprogramming and metabolic disturbances

Natural products, including TCM, have attracted increasing attention as potential interventions for kidney diseases (Pan et al., 2024; Shi J. et al., 2024; Amarasiri et al., 2025; Jiang et al., 2025; Miao et al., 2025). In recent years, accumulating evidence from basic studies has shown that natural products and TCM prescriptions can modulate metabolic reprogramming and restore metabolic homeostasis in podocytes through multiple signaling pathways, thereby preserving podocyte structure and function, reducing proteinuria, and slowing the decline in renal function.

### 4.1 Natural products

Extensive research has demonstrated that natural products play important roles in improving renal function and protecting against renal fibrosis (Cao et al., 2022; Ben-Azu et al., 2025; Liang et al., 2025; Miao and Vaziri, 2025; Liu et al., 2026c).

#### 4.1.1 Targeting energy metabolism

Targeting mitochondrial energy metabolism to regulate the utilization of metabolic substrates, such as glucose and fatty acids, is an important strategy for improving podocyte metabolism. Natural products can improve mitochondrial function by suppressing Drp1-mediated excessive mitochondrial fission. Berberine (BBR) is an alkaloid derived from plants, such as Coptis chinensis. In db/db mice and palmitic acid-treated mouse podocytes, berberine not only improved body weight, fasting blood glucose, and triglyceride levels in db/db mice but also attenuated excessive mitochondrial fission and dysfunction in podocytes by suppressing Drp1 expression and reducing its translocation to mitochondria, thereby protecting podocytes and ameliorating proteinuria in db/db mice (Qin et al., 2019). Polydatin has also been shown to suppress excessive mitochondrial fission and apoptosis in podocytes by downregulating Drp1 expression. It not only effectively reduced fasting blood glucose levels in KKAy mice but also ameliorated proteinuria and renal dysfunction. Furthermore, Drp1 expression was positively correlated with intracellular ROS levels, suggesting that polydatin may preserve mitochondrial function by limiting ROS production (Zheng et al., 2017). In addition to suppressing aberrant mitochondrial fission, several natural compounds and plant extracts can promote mitochondrial biogenesis through PGC-1α-related pathways. In db/db mice and palmitic acid-treated mouse podocytes, berberine may restore the activities of mitochondrial respiratory chain complexes I, IV, and V and citrate synthase and increase the expression of genes involved in FAO by activating the AMPK/PGC-1α signaling pathway, providing an additional mechanism of action beyond its effects on Drp1 expression. These effects may restore energy substrate utilization and ATP production, attenuate foot process effacement in diabetic mice, and significantly ameliorate proteinuria and renal dysfunction (Qin et al., 2020). Resveratrol, an agonist of SIRT1, attenuates high-glucose-induced podocyte injury and proteinuria in diabetic mice by activating the SIRT1/PGC-1α signaling pathway, upregulating Nrf1 and TFAM, restoring the activities of mitochondrial respiratory chain complexes I and III, and reducing mitochondrial ROS production. In addition, resveratrol can improve systemic glucose levels to some extent (Zhang T. et al., 2019). Li et al. found that astragaloside IV can also alleviate oxidative stress and mitochondrial dysfunction in podocytes induced by phenyl sulfate, a gut microbiota-derived metabolite, by activating the SIRT1/PGC-1α/Nrf1 signaling pathway (Li L. et al., 2024). In addition, salidroside could promote mitochondrial biogenesis, attenuate podocyte injury, and improve systemic metabolism and renal function in streptozotocin (STZ)-induced diabetic nephropathy (DN) mice, potentially through the SIRT1/PGC-1α axis (Xue et al., 2019). The R. procumbens n-butanol extract (J-NE) has also recently been shown to enhance mitochondrial biogenesis and preserve mitochondrial dynamics through the activation of SIRT1/PGC-1α (Ai et al., 2025). However, current studies on salidroside and J-NE remain limited to *in vivo* experiments and lack further validation in mechanistic *in vitro* studies.

Activation of Nrf2 to strengthen antioxidant defenses and mitochondrial biogenesis is another strategy for preserving mitochondrial function. Astragaloside IV promotes Nrf2 nuclear translocation by competitively binding to the amino acid sites on Keap1. It not only upregulates antioxidant genes to attenuate oxidative stress but also enhances mitochondrial biogenesis in podocytes by increasing TFAM expression, restoring electron transport chain complex activity, and increasing ATP production, thereby effectively reducing podocyte apoptosis and improving the urinary albumin-to-creatinine ratio (UACR) in diabetic mice (Shen et al., 2023). Astragaloside II has been shown to attenuate podocyte injury in diabetic rats. Su et al. found that astragaloside II increased the expression of mitophagy-related proteins by inhibiting Keap1 and upregulating Nrf2, thereby restoring mitophagy. Meanwhile, it increased the expression of the mitochondrial fusion protein Mfn2 and decreased that of the fission protein Fis1, restoring the mitochondrial dynamic balance and thereby attenuating podocyte injury in diabetic rats. The study also found that blood glucose levels did not differ significantly between astragaloside II- and losartan-treated diabetic rats, suggesting that its podocyte-protective effects may be independent of glucose lowering (Su et al., 2021).

#### 4.1.2 Targeting glucose metabolism

In addition to improving glucose utilization in podocytes by restoring the energy metabolic balance, natural products can also re-establish glucose metabolic homeostasis by suppressing abnormal metabolic shunts induced by high glucose levels. Ginsenoside Rb1 reduced abnormal sorbitol accumulation in podocytes under high-glucose conditions by binding to and inhibiting aldose reductase activity. It also decreased NOX4 expression and ROS production, improved mitochondrial function, and suppressed podocyte apoptosis. The study showed that its protective effect was greater than that of epalrestat, an aldose reductase inhibitor. Therefore, targeting the polyol pathway may represent an effective strategy by which TCM improves glucose metabolism in podocytes (He et al., 2022).

#### 4.1.3 Targeting amino acid metabolism

Research on TCM remains in its early stages in the field of amino acid metabolism. Metabolomic studies have shown that TCM prescriptions and natural compounds can ameliorate disturbances in amino acid metabolism and alleviate renal injury (Jin et al., 2026; Liu L. et al., 2026; Liu et al., 2026b). However, most existing evidence is based on metabolic alterations detected in the serum, urine, or kidney tissue and lacks podocyte-specific mechanistic validation. Whether TCM can improve glucose metabolic flux, reduce the abnormal accumulation of intermediate metabolites such as putrescine and BCAAs/BCKAs, and restore glutamine metabolic balance in podocytes requires further investigation. Therefore, the regulation of amino acid metabolic pathways may represent a promising approach for restoring metabolic homeostasis in podocytes.

#### 4.1.4 Targeting lipid metabolism

Natural products can restore lipid homeostasis in podocytes by targeting lipid uptake, efflux, synthesis, and oxidation (Guo et al., 2025). In addition to promoting FAO by improving mitochondrial energy metabolism, modulation of lipid transport has become another major focus of current research. For example, berberine not only increases the expression of FAO-related genes but also might reduce free fatty acid uptake by downregulating the fatty acid transporter CD36 (Qin et al., 2020). Morroniside can also ameliorate lipid metabolic disturbances by regulating lipid uptake and efflux. In KKAy mice and palmitic acid-or high glucose-treated mouse podocytes, morroniside upregulated PGC-1α expression, thereby activating PPARγ and suppressing CD36 to reduce lipid uptake, while upregulating LXRs/ABCA1 to enhance cholesterol and phospholipid efflux. These effects reduced lipid accumulation in podocytes, attenuated renal injury and lipid deposition in DN mice, and improved lipid profiles and renal function (Chen et al., 2024a). Curcumin reduced lipid accumulation in podocytes by binding to C-X-C chemokine ligand 16, thereby counteracting the downregulation of PPARγ and upregulation of COX2, and restoring Nrf2 nuclear translocation and transcriptional activity (Chen et al., 2024c). SIRT6 modulation may also be an effective strategy for limiting lipid accumulation. Wang et al. found that diosgenin reduced renal ectopic lipid accumulation and attenuated early podocyte injury in DN by activating SIRT6. This effect may be associated with the downregulation of downstream targets PDK4 and ANGPTL4, although the underlying mechanisms require further investigation (Wang Z. et al., 2022). In addition, Qin et al. recently demonstrated that four alkaloids isolated from *Alstonia scholaris* (L.) R. Br. alleviated oxidative stress and inflammation in podocytes, ameliorated lipid metabolic disturbances in DN mice, and reduced proteinuria, blood glucose levels, and lipid levels. Among these alkaloids, vallesamine has the strongest therapeutic effect. Further experiments suggested that vallesamine may modulate the PPARδ pathway and downstream gene expression. For example, it may enhance fatty acid transport and oxidation by upregulating ACSL1 and related targets, while reducing fatty acid synthesis by downregulating HMGCS2 and related targets. However, these regulatory mechanisms require further experimental validation (Qin et al., 2025).

#### 4.1.5 Targeting ion homeostasis

##### 4.1.5.1 Iron and ferroptosis

Natural products regulate the System Xc^−^/GSH/GPX4 axis through multiple mechanisms, restoring iron homeostasis and attenuating podocyte ferroptosis. Among these mechanisms, Nrf2 activation represents a major regulatory route. Triptolide suppresses podocyte ferroptosis and preserves cytoskeletal integrity by upregulating Nrf2 and subsequently increasing the expression of GPX4, SLC7A11, and FTH-1 (Wang H.-Q. et al., 2024). Similarly, swietenine, isolated from the fruits of Swietenia macrophylla King, promoted Nrf2 nuclear translocation through the Akt/GSK-3β/Nrf2 pathway and upregulated GPX4 and SLC7A11 expression, thereby reducing iron overload and lipid peroxidation and attenuating podocyte injury in STZ-induced DN rats (Duan et al., 2025). In addition to modulating Nrf2, astragaloside IV alleviated podocyte ferroptosis by increasing SLC7A11 and GPX4 expression via the MAPK14/SLC7A11/GPX4 axis (Qiu et al., 2026). Additionally, germacrone upregulated MMU_CircRNA_0000309 expression, which could bind to miR-188-3p and reduce its expression, thereby increasing GPX4 expression and modulating ferroptosis in a DN mouse model (Jin et al., 2022). However, most studies have examined the effects of natural products on ferroptosis through individual signaling pathways. In addition, evidence regarding the upstream regulation of iron metabolism, including iron uptake, transport, and storage, is limited. These mechanisms warrant further investigation in future studies.

##### 4.1.5.2 Calcium (Ca^2+^)

Studies on natural products and plant extracts targeting podocyte Ca^2+^ homeostasis have primarily focused on TRPC6-mediated Ca^2+^ influx. In podocytes exposed to high glucose or palmitic acid, astragaloside IV downregulated TRPC6 expression and reduced intracellular Ca^2+^ levels and the expression of B-cell lymphoma 2-associated X protein, thereby preventing apoptosis. This effect is likely associated with the inhibition of the TRPC6/CaN/NFAT signaling pathway (Yao et al., 2016; Zang et al., 2021). Tetrandrine inhibited Ca^2+^ influx by suppressing TRPC6-mediated RhoA/ROCK1 signaling and restoring nephrin and synaptopodin expression, thereby reducing foot process effacement and apoptosis and ameliorating proteinuria and renal dysfunction (Yu et al., 2020; Mao et al., 2022). The N-butanol extract of Rostellularia procumbens (L.) Nees also suppressed TRPC6 expression and reduced intracellular Ca^2+^ levels in podocytes (Ai et al., 2023). In addition, Atractylodes rhizoma water extract (ARE) upregulated synaptopodin expression and attenuated podocyte detachment, foot process effacement, and related injury in high-fructose-fed rats, possibly by indirectly inhibiting the TRPC6/p-CaMK4 signaling pathway through its antioxidant effects. However, the underlying mechanism requires further *in vitro* validation (Chen et al., 2021). TRPV1-mediated endoplasmic reticulum–mitochondrial Ca^2+^ transfer represents another potential regulatory target. Wei et al. found that capsaicin activated TRPV1 channels and induced a transient increase in intracellular Ca^2+^ levels. This increase subsequently enhanced the binding of the transcriptional repressor ZF5 to the Fundc1 promoter via the CaMKKβ–AMPK signaling axis, thereby downregulating Fundc1 expression and reducing MAM formation. These effects improve mitochondrial function and attenuate podocyte injury and proteinuria (Wei et al., 2020).

#### 4.1.6 Targeting immunometabolism

##### 4.1.6.1 NF-κB

NF-κB-related pathways are important targets for the modulation of podocyte immunometabolism by natural products. Multiple natural products can regulate NF-κB signaling via TLR4 (Liu H. et al., 2025). In STZ-induced DN rats and high glucose-stimulated podocytes, berberine inhibited NF-κB signaling by suppressing TLR4 expression, thereby reducing the production of inflammatory mediators, such as IL-1β, IL-6, and MCP-1, and attenuating podocyte apoptosis (Zhu et al., 2018). Similarly, astragalus polysaccharides (APS) exerted anti-inflammatory effects by downregulating the TLR4/NF-κB pathway (Guo M. et al., 2023). More recently, phillygenin was shown to exert both anti-inflammatory and anti-apoptotic effects by inhibiting the TLR4/MyD88/NF-κB pathway while concurrently activating the PI3K/Akt/GSK‐3β pathway (Feng et al., 2025). In addition, natural products can modulate NF-κB signaling by targeting upstream regulators other than TLR4. Wedelolactone acts on IκK to reduce the phosphorylation of IκB and NF-κB, thereby attenuating doxorubicin-induced oxidative stress and inflammatory responses in podocytes while restoring podocin expression (Zhu et al., 2019). Arctigenin exerts dual protective effects through PP2A activation. On the one hand, it induced dephosphorylation of NF-κB p65 and suppressed inflammation; on the other hand, it promoted dephosphorylation of T335 on Drebrin-1, thereby stabilizing the podocyte actin cytoskeleton (Zhong et al., 2019). Wogonoside attenuates high glucose-induced inflammatory responses and oxidative stress in podocytes by binding to NF-κB, thereby inhibiting NF-κB phosphorylation, nuclear translocation, and p65/p50 dimerization, and subsequently downregulating Mmp28 (Li X. et al., 2025). Barleriside A mitigated podocyte injury by inhibiting the NF-κB pathway and enhancing Nrf2 signaling (Li et al., 2024d). Moreover, Lycopi Herba extract (LHE) inhibited NF-κB phosphorylation and nuclear translocation. Through the NF-κB/TNF-α/STAT3 axis, LHE reduced inflammatory mediators such as TNF-α, IL-1β, and IL-6, thereby attenuating high glucose-induced inflammatory injury in podocytes (Chen et al., 2026).

##### 4.1.6.2 NLRP3 inflammasome and pyroptosis

Targeting the NLRP3 inflammasome and podocyte pyroptosis is a promising therapeutic strategy. Astragaloside IV inhibited the expression of NLRP3, caspase-1, and inflammatory cytokines in the podocytes of db/db mice (Feng et al., 2021). He et al. further found that astragaloside IV could suppress NLRP3 inflammasome activation by upregulating Klotho expression, thereby reducing ROS accumulation and NF-κB activation (He et al., 2023). AMPK also plays a key role in mediating anti-inflammatory effects. Geniposide and genipin-1-β-D-gentiobioside (GG), both derived from Gardenia jasminoides Ellis, exerted anti-pyroptotic effects by activating the AMPK/SIRT1 signaling pathway and suppressing downstream NF-κB/NLRP3 signaling (Li F. et al., 2020; Li et al., 2021). Fucoidan has also been reported to inhibit NLRP3 inflammasome activation through the AMPK/mTORC1 axis (Wang M.-Z. et al., 2022). In addition, triptolide reduced ROS production by activating the Nrf2/HO-1 signaling pathway, thereby suppressing the NLRP3 inflammasome and attenuating pyroptosis (Lv et al., 2023). Total flavones of Abelmoschus manihot modulate podocyte pyroptosis at the epigenetic level. They increased m6A modification of PTEN mRNA by upregulating METTL3-dependent m6A RNA methylation, thereby suppressing PTEN expression and activating the PI3K/Akt signaling pathway, which in turn inhibited the NLRP3 inflammasome activation (Liu et al., 2021).

In summary, current studies indicate that natural products and plant extracts can regulate multiple metabolic processes and restore metabolic homeostasis in the podocytes. Animal studies have shown the potential of these drugs to reduce proteinuria and improve renal function, whereas *in vitro* studies have demonstrated varying degrees of protection against podocyte injury. Moreover, individual natural products may simultaneously modulate multiple metabolic processes, reflecting the multi-target and multi-pathway regulatory properties of TCM (Table 1). Taking Astragalus membranaceus and its representative constituents as an example, Astragalus membranaceus is widely used in the prevention and treatment of kidney diseases (Shi Y. et al., 2024). Multiple natural products derived from Astragalus membranaceus can target Nrf2 and the SIRT1/PGC-1α/Nrf1 pathway to improve energy metabolism and regulate SLC7A11, GPX4, and TRPC6 expression to restore ion homeostasis. Moreover, they exert anti-inflammatory effects through NF-κB and NLRP3 signaling. In addition, BBR acts on multiple targets, including the AMPK signaling pathway, NF-κB, and Nrf2, to attenuate podocyte injury. However, the available evidence should be interpreted cautiously, and several issues warrant consideration. Doses used in animals cannot be directly extrapolated to humans without pharmacokinetic evaluation and appropriate conversion to human-equivalent doses. In models of diabetes or dyslipidemia, it also remains unclear whether improvements in proteinuria and renal function are due to direct effects on podocytes or are secondary to systemic glucose- or lipid-lowering effects. In addition, although experiments in cultured podocytes provide direct evidence at the cellular level, most *in vivo* studies lack podocyte-specific genetic target validation.

**TABLE 1:** Mechanisms of natural products in regulating podocyte metabolism.

Compounds	Sources	Model type	Animal/cell models	Doses/concentrations	Molecular mechanisms	Causal intervention	Metabolic pathway	Ref
Astragaloside II	*Astragalus membranaceus*	*In vivo*	STZ-induced diabetic rats	3.2, 6.4 mg/kg	Upregulating expression of Nrf2	—	energy metabolism	(Su et al., 2021)
Astragaloside IV	*Astragalus membranaceus*	*In vivo + in vitro*	STZ-induced diabetic mice and HG-induced mouse podocytes	6 mg/kg; 3, 10, 20, 40, 80 μM	Activating Nrf2-ARE/TFAM pathway	Nrf2 inhibitor (trigonelline) and Nrf2 siRNA; TFAM siRNA	energy metabolism	(Shen et al., 2023)
Astragaloside IV	*Astragalus membranaceus*	*In vivo + in vitro*	db/db mice and phenyl sulfate-induced MPCs	20 mg/kg; 50, 100 μM	Activating SIRT1/PGC-1α/Nrf1 pathway	SIRT1 inhibitor (EX527)	energy metabolism	(Li et al., 2024a)
Astragaloside IV	*Astragalus membranaceus*	*In vivo + in vitro*	db/db mice and HG-induced MPC-5 cells	1 g/kg; 30 μM	Upregulating expression of SLC7A11 and GPX4 by inhibiting MAPK14	p38 MAPK activator (C16-PAF)	Iron and Ferroptosis	(Qiu et al., 2026)
Astragaloside IV	*Astragalus membranaceus*	*In vitro*	HG-induced mouse podocytes	10, 20, 40 µM	Downregulating expression of TRPC6 and suppressing intracellular Ca^2+^ levels	—	Ca^2+^ Signaling	(Yao et al., 2016)
Astragaloside IV	*Astragalus membranaceus*	*In vitro*	Palmitic acid-induced mouse podocyte cell lines	10, 20, 40, 80 μM	Downregulating expression of TRPC6 and suppressing intracellular Ca^2+^ levels	Ca^2+^ scavenger (BAPTA) and Ca^2+^ ionophore (ionomycin); TRPC6 antagonist (SKF96365), TRPC6 agonist (OAG) and TRPC6 siRNA	Ca^2+^ Signaling	(Zang et al., 2021)
Astragaloside IV	*Astragalus membranaceus*	*In vivo + in vitro*	db/db mice and HG-induced mouse podocytes	40 mg/kg; 10, 20, 40 µM	Inhibiting NLRP3 inflammasome-mediated inflammation	NLRP3 overexpression	Immunometabolism	(Feng et al., 2021)
Astragaloside IV	*Astragalus membranaceus*	*In vivo + in vitro*	High-fat diet + STZ-induced DKD rats and HG-induced mouse podocytes	40, 80 mg/kg; 50, 75, 100 μM	Upregulating expression of endogenous klotho protein and inhibiting NF-κB/NLRP3-induced pyroptosis	klotho overexpression and klotho siRNA	Immunometabolism	(He et al., 2023)
APS	*Astragalus membranaceus*	*In vivo + in vitro*	STZ-induced DKD rats and HG-induced MPC5 cells	200, 400, 800 mg/kg; 5% APS serum	Decreasing production of inflammatory cytokines via inhibiting TLR4/NF-κB pathway	—	Immunometabolism	(Guo et al., 2023a)
BBR	*Coptis chinensis*, *etc.*	*In vivo + in vitro*	db/db mice and palmitic acid-induced mouse podocytes	300 mg/kg; 0.4 μM	Inhibiting Drp1 expression and translocation to mitochondria	Drp1 overexpression and Drp1 siRNA	energy metabolism	(Qin et al., 2019)
BBR	*Coptis chinensis*, *etc.*	*In vivo + in vitro*	db/db mice and palmitic acid-induced mouse podocytes	200, 300 mg/kg; 0.4 μM	Activating AMPK/PGC-1α pathway, downregulating expression of CD36	AMPK inhibitor (Compound C); PGC-1α siRNA	energy metabolism, Lipid Metabolism	(Qin et al., 2020)
BBR	*Coptidis rhizome*, *Cortex phellodendri* and *Berberis vulgaris*	*In vivo + in vitro*	STZ-induced DKD rats and HG- induced mouse podocytes	50, 100, 200 mg/kg; 10, 30, 90 μM	Inhibiting TLR4/NF-κB pathway	—	Immunometabolism	(Zhu et al., 2018)
Polydatin	*Polygonum cuspidatum*	*In vivo + in vitro*	KKAy mice and HG-induced MPC-5 cells	100 mg/kg; 25 mM	Inhibiting ROS production and downregulating expression of Drp1	Drp1 overexpression and Drp1 siRNA	energy metabolism	(Zheng et al., 2017)
Resveratrol	*Polygonum cuspidatum*, *etc.*	*In vivo + in vitro*	STZ-induced diabetic mice and HG-induced mouse podocytes	30 mg/kg; 10 μM	Activating SIRT1/PGC-1α pathway	—	energy metabolism	(Zhang et al., 2019a)
N-butanol extract	*Rostellularia procumbens* (L) Nees	*In vivo*	Adriamycin-induced mice	1.0, 2.0, 4.0 g/kg	Activating SIRT1/PGC-1α pathway	—	energy metabolism	(Ai et al., 2025)
N-butanol extract	*Rostellularia procumbens* (L) Nees	*In vivo + in vitro*	Adriamycin-induced mice and adriamycin-induced MPC5 cells	0.5, 1.0, 2.0 g/kg; 10 μg/mL	Downregulating expression of TRPC6 and suppressing intracellular Ca^2+^ levels, reducing inflammatory factors such as TNF-α and IL-6	—	Ca ^2+^ Signaling, Immunometabolism	(Ai et al., 2023)
Geniposide	*Gardenia jasminoides* Ellis	*In vivo + in vitro*	High-fat diet + STZ-induced DKD mice and HG-induced mouse podocytes	25, 50 mg/kg; 50, 100, 200 μg/mL	Activating AMPK/SIRT1 pathway, subsequently inhibiting NF-κB and NLRP3	AMPK siRNA	Immunometabolism	(Li et al., 2020a)
GG	*Gardenia jasminoides* Ellis	*In vivo + in vitro*	High-fat diet + STZ-induced DKD mice and HG-induced mouse podocytes	25, 50 mg/kg; 5, 10, 20 μM	Activating AMPK/SIRT1 pathway, thereby inhibiting NF-κB and NLRP3	AMPK siRNA	Immunometabolism	(Li et al., 2021)
Triptolide	*Tripterygium wilfordii* Hook F	*In vivo + in vitro*	db/db mice and HG + palmitic acid-induced HPCs	50 μg/kg; 5 nM	Upregulating expression of GPX4, SLC7A11, and FTH-1 and downregulating expression of TFR-1 via activating Nrf2	Nrf2 inhibitor (ML385)	Iron and Ferroptosis	(Wang et al., 2024b)
Triptolide	*Tripterygium wilfordii* Hook F	*In vivo + in vitro*	High-fat diet + STZ-induced DKD mice and HG-induced MPC5 cells	100 μg/kg; 10 μM	Activating Nrf2/HO-1 pathway and inhibiting NLRP3 inflammasome pathway	Nrf2 inhibitor (ML385) and Nrf2 siRNA	Immunometabolism	(Lv et al., 2023)
Salidroside	*Rhodiola rosea* L	*In vivo*	High-fat diet + STZ-induced DKD mice	50, 100 mg/kg	Activating SIRT1/PGC-1α pathway	—	energy metabolism	(Xue et al., 2019)
Ginsenoside Rb1	*Panax notoginseng*	*In vivo + in vitro*	STZ-induced DKD mice and HG-induced MPCs	40 mg/kg; 0.1, 1, 10 µM	Inhibiting AR activity and downregulating expression of NOX4	—	Glucose Metabolism	(He et al., 2022)
Morroniside	*Cornus officinalis*	*In vivo + in vitro*	KKAy mice and HG/palmitic acid-induced mouse podocytes	50 mg/kg; 0.5, 1, 2 μM	Upregulating expression of PGC-1α, PPARγ, LXRs, and ABCA1, downregulating expression of CD36	PGC-1α siRNA	Lipid Metabolism	(Chen et al., 2024a)
Curcumin	*Curcuma longa*	*In vivo + in vitro*	db/db mice and HG/palmitic acid‐induced human podocytes, recombinant CXCL16‐treated human podocytes	160 mg/kg; 10 µM	Inhibiting CXCL16-induced downregulation of PPARγ and upregulation of COX2, upregulating expression of Nrf2	CXCL16 siRNA	Lipid Metabolism	(Chen et al., 2024c)
Diosgenin	Leguminosae and Dioscoreaceae plants	*In vivo + in vitro*	db/db mice and palmitic acid-induced MPC5 cells	30, 90 mg/kg; 0.1, 1 μM	Regulating SIRT6	SIRT6 inhibitor (OSS-128167) and SIRT6 agonist (MDL800)	Lipid Metabolism	(Wang et al., 2022b)
Alkaloids (picrinine, scholaricine, 19-epi-scholaricine and vallesamine)	*Alstonia scholaris* (L.) R. Br	*In vivo + in vitro*	High-fat diet + STZ-induced DKD mice and HG-induced MPC5 cells	total alkaloids 10, 20 mg/kg, picrinine 10 mg/kg; 5 μM, scholarcine 2.5 mg/kg; 1 μM, 19-epi-scholarcine 1 mg/kg; 1 μM, vallesamine 5 mg/kg; 10 μM	Upregulating expression of PPARδ and modulating expression of downstream genes	—	Lipid Metabolism	(Qin et al., 2025)
Swietenine	*Swietenia macrophylla* King	*In vivo + in vitro*	High-fat diet + STZ-induced DKD rats and HG-induced MPC5 cells	25, 50 mg/kg; 1, 5, 10 μM	Activating Akt/GSK-3β/Nrf2 pathway, upregulating expression of GPX4 and SLC7A11	Akt inhibitor (MK-2206); GSK-3β inhibitor (LY2090314); Nrf2 inhibitor (ML385)	Iron and Ferroptosis	(Duan et al., 2025)
Germacrone	*Rhizoma Curcuma*	*In vivo + in vitro*	db/db mice and HG-induced MPC5 cells	10 mg/kg;/	Upregulating expression of GPX4 via mmu_circRNA_0000309/miR-188-3p/GPX4 axis	mmu_circRNA_0000309 shRNA; miR-188-3p mimic; GPX4 overexpression	Iron and Ferroptosis	(Jin et al., 2022)
Tetrandrine	*Stephania tetrandra*	*In vitro*	TRPC6-overexpressing MPC5 cells	10 μM	Downregulating expression of TRPC6, suppressing intracellular Ca^2+^ levels and inhibiting RhoA/ROCK pathway	—	Ca ^2+^ Signaling	(Yu et al., 2020)
Tetrandrine	*Stephania tetrandra*	*In vivo + in vitro*	Uninephrectomy + adriamycin-induced rats and TRPC6-overexpressing MPC5 cells	4, 8, 16 mg/kg; 40 μM	Downregulating expression of TRPC6, suppressing intracellular Ca^2+^ levels and inhibiting RhoA/ROCK pathway	—	Ca ^2+^ Signaling	(Mao et al., 2022)
ARE	*Atractylodis rhizoma*	*In vivo*	Fructose-induced rats	720, 1090 mg/kg	Inhibiting TRPC6/p-CaMK4 pathway	—	Ca ^2+^ Signaling	(Chen et al., 2021)
Capsaicin	*Capsicum genus*	*In vivo + in vitro*	db/db mice/STZ-induced diabetic mice and HG-induced mouse podocytes	normal chow containing 0.01% capsaicin; 1 μM	Activating TRPV1 and reducing MAM formation	TRPV1 knockout and TRPV1 blocker (iRTX)	Ca ^2+^ Signaling	(Wei et al., 2020)
Phillygenin	*Forsythia suspensa*	*In vivo + in vitro*	db/db mice and HG-induced MPCs	20, 50 mg/kg; 10, 20, 40, 80, 160, 320 μM	Downregulating expression of inflammatory cytokines and inhibiting TLR4/MyD88/NF-κB pathway	TLR4 agonist (RS09); PI3K inhibitor (WORT)	Immunometabolism	(Feng et al., 2025)
Wedelolactone	*Eclipta prostrata*	*In vitro*	Doxorubicin-induced MPC-5 cells	1.25, 5, 20 μM	Downregulating expression of inflammatory cytokines via inhibiting phosphorylation of IκK/IκB/NF-κB pathway	—	Immunometabolism	(Zhu et al., 2019)
Arctigenin	*Fructus Arctii*	*In vivo + in vitro*	db/db mice, STZ-induced diabetic mice and HG -induced human podocytes	40 mg/kg; 10 μM	Activating PP2A and inhibiting NF-κB phosphorylation	Podocyte-specific PP2A knockout	Immunometabolism	(Zhong et al., 2019)
Wogonoside	*Scutellaria baicalensis*	*In vivo + in vitro*	High-fat diet + STZ-induced DKD mice and HG-induced MPC5 cells	12.5, 25, 50 mg/kg; 10, 20, 40 μM	Inhibiting NF-κB p65 phosphorylation, nuclear translocation and dimerization with p50, thereby downregulating expression of Mmp28	p65 activator (TNF-α) and p65 inhibitor (SC75741), p65 siRNA and p65 overexpression	Immunometabolism	(Li et al., 2025b)
Barleriside A	*Plantaginis semen*	*In vivo + in vitro*	5/6 nephrectomized rats and ZAS-stimulated MPC5 cells	10 mg/kg; 20 μM	Inhibiting IκB/NF-κB pathway and enhancing Keap1/Nrf2 pathway via suppressing aryl hydrocarbon receptor signaling	AHR inhibitor (CH223191) and AHR siRNA	Immunometabolism	(Li et al., 2024d)
LHE including ursolic acid, apigenin and quercetin	*Lycopi Herba*	*In vivo + in vitro*	STZ-induced diabetic mice and HG-induced HPCs	50, 100 mg/kg; 50, 100 μg/mL	Inhibiting NF-κB/TNF-α/STAT3 axis via suppressing the phosphorylation and nuclear translocation of NF-κB and STAT3	—	Immunometabolism	(Chen et al., 2026)
Fucoidan	*Laminaria japonica*	*In vivo + in vitro*	Uninephrectomy + high-fat diet + STZ-induced DKD rats and HG-induced MPC-5 cells	120 mg/kg; 20 μg/mL	Inhibiting NLRP3 inflammasome via AMPK/mTORC1/NLRP3 axis	—	Immunometabolism	(Wang et al., 2022a)
Total flavones of Abelmoschus manihot	*Abelmoschus manihot*	*In vitro*	HG-induced MPC-5 cells	20 μg/mL	Inhibiting NLRP3 inflammasome by promoting METTL3-dependent m6A modification	METTL3 siRNA and METTL3 overexpression	Immunometabolism	(Liu et al., 2021)

ABCA1, ATP‐binding cassette transporter A1; AHR, aryl hydrocarbon receptor; Akt, protein kinase B; AMPK, AMP‐activated protein kinase; APS, astragalus polysaccharides; AR, aldose reductase; ARE, atractylodes rhizoma water extract; BBR, berberine; CaMK4, Ca^2^^+^/calmodulin‐dependent protein kinase 4; COX2, cyclooxygenase 2; CXCL16, C‐X‐C chemokine ligand 16; DKD, diabetic kidney disease; Drp1, dynamin‐related protein 1; FTH‐1, ferritin heavy chain 1; GG, genipin‐1‐β‐D‐gentiobioside; GPX4, glutathione peroxidase 4; GSK‐3β, glycogen synthase kinase‐3β; HG, high glucose; HO‐1, heme oxygenase‐1; IL‐6, interleukin‐6; IκB, inhibitor of κB; IκK, IκB kinase; Keap1, kelch‐like ECH‐associated protein 1; LHE, Lycopi Herba extract; LXR, liver X receptor; m6A, N6‐methyladenosine; MAM, mitochondria‐associated endoplasmic reticulum membrane; MAPK, mitogen‐activated protein kinase; METTL3, methyltransferase‐like 3; mTORC1, mechanistic target of rapamycin complex 1; MyD88, myeloid differentiation factor‐88; NF‐κB, nuclear factor‐κB; NLRP3, NOD‐like receptor family pyrin domain‐containing protein 3; NOX4, nicotinamide adenine dinucleotide phosphate oxidase 4; Nrf1, nuclear respiratory factor 1; Nrf2, nuclear factor erythroid 2‐related factor 2; PGC‐1α, peroxisome proliferator‐activated receptor‐γ coactivator‐1α; PI3K, phosphatidylinositol 3‐kinase; PP2A, protein phosphatase 2A; PPARγ, peroxisome proliferator‐activated receptor γ; PPARδ, peroxisome proliferator‐activated receptor δ; RhoA, ras homolog family member A; ROCK, Rho‐associated coiled‐coil containing protein kinase; ROS, reactive oxygen species; SIRT1, sirtuin 1; SIRT6, sirtuin 6; SLC7A11, solute carrier family 7 member 11; STAT3, signal transducer and activator of transcription 3; STZ, streptozotocin; TFAM, transcription factor A, mitochondrial; TFR‐1, transferrin receptor 1; TLR4, toll-like receptor 4; TNF‐α, tumor necrosis factor‐α; TRPC6, transient receptor potential channel C6; TRPV1, transient receptor potential cation channel subfamily V member 1; ZAS, zymosan‐activated serum.

### 4.2 TCM prescriptions

Several TCM prescriptions have shown potential renal benefits in small clinical studies of patients with kidney disease. For example, Modified Hu-lu-ba-wan showed beneficial effects in reducing proteinuria in patients with early-stage DKD (Gong et al., 2021). The BaoShenTongLuo formula has been shown in clinical studies to improve UACR and renal function in patients with DKD while reducing urinary nephrin excretion, suggesting that it may reduce proteinuria and delay DKD progression by alleviating podocyte injury (Shen et al., 2020; Cui et al., 2024). Danggui Buxue Decoction has also reported renal benefits in clinical trials involving patients with DKD, including reductions in urinary protein excretion and oxidative stress, and improvements in renal function (Zheng et al., 2023). TCM prescriptions have also shown favorable renal effects and acceptable safety profiles in clinical trials when combined with agents such as SGLT2 inhibitors and ACEIs/ARBs. For example, Jinlida granules combined with dapagliflozin effectively improved lipid metabolism and renal function, with greater efficacy than that observed in the control group (Xia et al., 2025). Overall, existing clinical studies provide preliminary evidence that TCM prescriptions may reduce proteinuria and slow the progression of renal dysfunction. As discussed below, further preclinical studies suggest that multiple TCM prescriptions exert nephroprotective effects by modulating podocyte metabolism.

In terms of energy metabolism, Modified Hu-lu-ba-wan increased the expression of optic atrophy 1 by activating the PKM2/PGC-1α signaling pathway, thereby promoting mitochondrial fusion and restoring mitochondrial dynamic balance in podocytes (Gong et al., 2024). The BaoShenTongLuo formula restored mtDNA copy number and the activities of mitochondrial respiratory chain complexes I, III, and IV by regulating AMPK signaling and upregulating PGC-1α and TFAM. Moreover, it increases Mfn2 expression, thereby improving mitochondrial biogenesis and dynamics and attenuating podocyte injury (Guo Y. et al., 2023). Jinlida granules also activated the AMPK/PGC-1α pathway, thereby suppressing Drp1-mediated excessive mitochondrial fission, ameliorating mitochondrial dysfunction and oxidative stress, and reducing podocyte apoptosis (Sun et al., 2025).

In terms of lipid metabolism, Gandi Capsule downregulated the transcription factor hepatocyte nuclear factor 4-α by activating the SIRT1/AMPK signaling axis, thereby reducing lipid accumulation in podocytes and urinary albumin excretion in db/db mice (Zhang et al., 2022). Wenyang Jiedu Tongluo formula upregulated SIRT1 by inhibiting JAML, thereby downregulating SREBP1 and fatty acid synthesis-related enzymes, including ACC1, FASN, and SCD1, improving podocyte lipid metabolism, and reducing glomerular lipid deposition (Liu Y. et al., 2025). In addition, Wang et al. identified 37 lipid biomarkers of DKD through lipidomic analysis and found that Danggui Buxue Decoction significantly restored the levels of triglyceride-related markers and cardiolipins, thereby ameliorating disturbances in triglyceride and cardiolipin metabolism. *In vitro* experiments further confirmed that Danggui Buxue Decoction and its major active components upregulated SIRT1, PPARG, and ABCA1 expression and significantly reduced lipid deposition in podocytes (Wang X. et al., 2025).

In terms of ion homeostasis, TCM prescriptions primarily exert antioxidant and anti-ferroptotic effects by modulating the GPX4/SLC7A11 axis in the podocytes. The Sanqi qushi formula upregulated GPX4 expression by inhibiting JNK1 and reducing FoxO1 nuclear translocation, thereby suppressing ferroptosis of podocytes and improving renal function in Passive Heymann nephritis rats (Li H. et al., 2026). The Qing-Re-xiao-zheng-(yi-qi) formula inhibited podocyte ferroptosis by upregulating GPX4/SLC7A11 and downregulating ACSL4 through AMPK signaling activation (Zhang et al., 2025c). Yitangkang decoction also activated AMPK and enhanced SLC7A11 palmitoylation at Cys327 through the AMPKα1/ZDHHC8 axis, thereby stabilizing SLC7A11 protein, upregulating GPX4, and protecting podocytes against ferroptosis (Yu et al., 2026). Studies on the regulation of Ca^2+^ by TCM prescriptions have primarily focused on the TRPC6 channel. Tangshen Formula inhibited TRPC6-mediated Ca^2+^ influx and restored the expression of Talin1, integrin β1, and paxillin, thereby reducing podocyte migration, actin cytoskeletal remodeling, foot process effacement, and alleviating proteinuria in db/db mice (Wang et al., 2020). Qian yang yu yin granule was also shown to inhibit TRPC6-mediated Ca^2+^ influx, thereby correcting abnormal autophagy through the TRPC6-CaMKKβ-AMPK-mTOR pathway and ameliorating hypertensive renal injury (Ma et al., 2023).

In terms of immunometabolism, Xuebijing injection was found to suppress the expression of TNF-α, IL-1β, and IL-6, thereby attenuating inflammatory injury, restoring nephrin expression, and ameliorating proteinuria in mice with adriamycin-induced nephrotic syndrome (Yuan et al., 2024). A recent study demonstrated that oxidative stress and inflammatory responses are involved in idiopathic membranous nephropathy (Wang et al., 2023a). Zhen-wu-tang attenuated podocyte inflammatory responses by suppressing the NF-κB pathway and NLRP3 inflammasome, thereby reducing proteinuria and renal injury in rats with membranous nephropathy (MN) (Liu B. et al., 2019). Additionally, Shenkangning capsule inhibited NF-κB-mediated inflammatory responses while activating Nrf2-dependent antioxidant defenses, thereby synergistically protecting podocytes and attenuating podocyte injury in mice with focal segmental glomerulosclerosis (Wei et al., 2022).

## 5 Limitations and perspectives

Although accumulating evidence suggests that podocyte metabolic reprogramming and disruption of metabolic homeostasis are important contributors to podocyte injury and detachment, several issues in this field remain unclear. It should be noted that most of the mechanistic evidence discussed in this review is derived from rodent models and immortalized podocyte cell lines, with current research primarily focusing on DKD. The extent to which these findings translate to human podocytes or to other glomerular diseases, including focal segmental glomerulosclerosis, MN, and LN, remains insufficiently established. Therefore, future studies should characterize the disease-specific metabolic features of podocytes across different glomerular diseases to facilitate the development of tailored therapeutic strategies. Metabolism represents a complex and interconnected network in which individual pathways influence each other and are further shaped by diverse external factors. Across different studies, alterations in podocyte glycolysis, mitochondrial oxidative phosphorylation, amino acid metabolism, and lipid metabolism have not been consistent. These discrepancies may reflect differences in disease type, model source, cellular differentiation status, stimulus intensity, intervention duration, and other experimental conditions used in the studies. Therefore, the dynamic changes in podocyte metabolism across different disease contexts and stages, microenvironmental conditions, and energy demands warrant further investigation. Given the complexity of cellular metabolism, metabolic intervention should not be viewed simply as the activation or inhibition of a single target or pathway. For example, studies on podocyte-specific PGC-1α regulation have shown that PGC-1α overexpression in podocytes may induce a collapsing glomerulopathy-like phenotype, suggesting that this target may have a relatively narrow therapeutic window (Li et al., 2017). Therefore, defining the appropriate range of modulation for individual metabolic targets and restoring metabolic balance within this range are crucial.

Research on the targeted regulation of podocyte metabolism by TCM also has several limitations. Although current studies have demonstrated favorable podocyte-protective effects, some lack validation using podocyte-specific gene knockout or targeted interventions. Moreover, many studies remain limited to correlative observations or validation of individual targets without further delineation of the complete upstream and downstream mechanisms. In addition, most current evidence is derived primarily from cell and animal studies, whereas clinical evidence remains limited. Although existing clinical studies suggest potential benefits, they commonly have limitations, such as small sample sizes, single-center designs, inadequate quality control, and short follow-up periods. High-quality, large-scale, randomized controlled trials are urgently needed to confirm these observations. Many natural products have not undergone systematic safety evaluation. Their clinical use faces multiple safety challenges, including batch-to-batch variability in bioactive constituents, potential herb-drug interactions, and the intrinsic toxicity of certain constituents. Therefore, the safety of natural products remains an important issue that requires careful evaluation in future clinical trials. The limited bioavailability of active TCM components and insufficient *in vivo* delivery efficiency also constrain their clinical application. For example, berberine has low oral bioavailability and limited absorption (Khoshandam et al., 2022), whereas curcumin is characterized by poor absorption, rapid metabolism, and fast systemic clearance (Tabanelli et al., 2021). For podocytes, drug delivery is further constrained by the structural characteristics of the glomerular filtration barrier. As podocytes are located outside the glomerular capillary lumen, local drug exposure may be limited. Therefore, future studies should focus on nanocarriers and podocyte-specific delivery strategies to enhance the *in vivo* efficacy of active TCM components.

Given the complex clinical phenotypes and diverse etiologies of kidney diseases, network medicine provides a suitable framework for exploring novel mechanisms and identifying therapeutic targets. Technologies such as single-cell and single-nucleus transcriptomics, spatial metabolomics, and proteomics can facilitate the investigation of metabolic mechanisms and intercellular interactions within the kidney and help identify novel biomarkers and potential therapeutic targets. In the future, advanced omics technologies may enable precision treatment based on the specific metabolic profiles of individual patients (Pandey and Loscalzo, 2023; Sharma et al., 2026). Systems pharmacology can be used to identify the key active components of TCM formulations and their associated targets (Zhang W. et al., 2019). By integrating multi-omics data and elucidating the complex relationships among TCM components, molecular targets, mechanistic pathways, and clinical disease phenotypes, systems pharmacology may facilitate the transition of TCM from empirical interventions toward precision medicine.

## 6 Conclusion

Podocyte metabolism is a complex dynamic process. With continued advances in this field, the mechanisms underlying podocyte metabolic reprogramming and homeostasis have become increasingly well characterized. Growing evidence has revealed that microenvironmental alterations, including hyperglycemia, lipid overload, and mechanical stress, reshape glucose, amino acid, and lipid utilization, promote the accumulation of metabolic intermediates, disrupt ion homeostasis, and increase oxidative stress. These metabolic disturbances further activate innate immune pathways, contributing to podocyte injury. Therefore, restoring the balance of glucose, amino acid, and lipid metabolism, maintaining ion homeostasis, and suppressing aberrant immunometabolic activation may be important strategies for alleviating podocyte injury and slowing the progression of glomerular diseases. Regarding TCM-based interventions, natural products have been shown to attenuate podocyte injury and ameliorate proteinuria and renal dysfunction by modulating podocyte metabolism in diverse preclinical models, including DKD and MN, offering new perspectives for the prevention and treatment of podocyte injury. Additionally, emerging mechanisms, such as those involving amino acid metabolism, offer new opportunities for identifying potential targets of TCM interventions. Together with the limitations and future directions outlined in Section 5, these insights may help guide further mechanistic and translational research on TCM-based interventions targeting podocyte metabolism. Given that the current evidence remains predominantly preclinical and high-quality clinical validation is limited, more rigorous mechanistic studies and targeted clinical trials are needed to advance precision treatment for podocyte-related glomerular diseases.
